# Remarks on Some of the Proceedings of the New-York State Medical Society, at the Annual Meeting, Feb. 1, 1846

**Published:** 1846-10

**Authors:** 


*Remarks on some of the Proceedings of the New-York State Medical Society, at the annual meeting, Feb.* 1, 1846. BY CHARLES B. COVENTRY, M. D.

The following article we take from the Sept. No. of the New-York Journal of Medicine. It relates to subjects, which, at the present time, are of interest to the profession, and upon which the County Societies throughout the State are invited to take action for the guidance of the State Society, at its next annual meeting. We commend the article to the consideration of our readers.—*Editor Buffalo Journal.*

"On looking over the "Transactions of the Medical Society of the State of New-York" for 1846, I was surprised to find that certain resolutions which were appended to a report made to the society the previous year by N. S. Davis, as a chairman of the committee, were adopted by the society, and a committee appointed to submit them to the county societies for their approbation. The two important resolutions, and the only ones which I shall notice, are the following:

1. *Resolved*, That every candidate for license to practice physic and surgery should be required to give evidence to the board by whom he is examined, that he has received a good academical education, and that he has studied physic and surgery not less four years with some regular licensed physician: that he possesses a competent knowledge of physic and surgery; that he has personally assisted in the dissection of at least two good anatomical subjects under the direction of a regularly educated physician, or in some incorporated medical College in this State; that he be practically acquainted with botany, and has presented the board with an essay on some medical subject written by himself; and that he is twenty-one years of age, and of good moral character.

2. *Resolved*, That a board of censors, to consist of not more than five from each of the censoria' districts, be chosen, the choice to be made from candidates who may be nominated by the county medical societies composing the respective censorial districts, by a majority of the votes of all the members of the State Medical Society present and voting at the annual meeting thereof. The term of office of each censor in the respective censorial districts to be determined at the first election by lot, and thereafter the vacancy of one in each censorial district to be filled by the State Society at its annual meeting; seven of said court of censors to constitute a quorum for the transaction of business, to meet at such times and places, and be governed by such rules and regulations, and receive such compensation for their services as the State Medical Society shall from time to time direct. The board of censors there constituted shall examine all candidates for licenses to practice physic and surgery, and to such as are found qualified agreeably to the requirements of preceding resolution, it shall be their duty to grant a license to practice physic and surgery under the auspices of the society, after filing their certificates as required by law. Said board may also examine such candidates for the degree of Doctor of Medicine as have been practitioners of medicine in good standing for a least five years, and by a vote of two-thirds of the board of examiners present, may recommend such candidates to the Regents of the University as suitable persons on whom to confer said degree. Each person licensed, to pay to the president of the board of censors, for the use of the society, twenty-five dollars; and each person who is recommended as entitled to t e degree of Doctor of Medicine, to pay as aforesaid the sum of thirty-five dollars, and no license to be granted or degree conferred, until said fees are paid. And that said board annually report their doings to this body, and all laws relating to granting licenses to practice physic and surgery, and to conferring the degree of Doctor of Medicine, in this State, inconsistent with the above regulations, to be repealed.

The above resolutions having been adopted by the State society, and submitted to the county societies for their approbation, it is reasonable to infer, that if sanctioned by the county societies, they are to form the basis of application for future legislation, and are expressive of the sentiments of the State society. The ostensible object is *medical reform:* but the county societies, and the individual members of the professsion, may ask, why we are called upon to abandon a system which has been in successful operation for forty years: a system, by which the profession has been elevated from a state of chaos to its present high position, and under which the profession has gone on improving;—one in which the qualification of those admitted to the profession depend upon the profession itself.

As the resolutions themselves contain no reasons for such important changes, we are compelled to seek for them in the report of the chairman of the committee. Here we find an abundance of assertions as to the present degraded condition of the profession, and the facilities with which licenses are procured. Now these assertions may or may not be true: they certainly are not self-evident propositions, and we have searched in vain in the report for any proof of their truth. If they are not true, the semi-official endorsement of the State society, by adopting the resolutions, can give them no additional weight. We will, however, examine the reasons in the order in which they are presented. The report says:

"What then are the evils of which we complain? We answer—first, there is no uniformity in the standard of attainments required of the candidates for admission into the profession. These numerous boards contain more than 250 physicians. Many, if not most of them, are chosen by their respective societies, more on account of the convenience with which they can come together, than for their fitness for the important station. While one board may be faithful in the discharge of its duties, another may be lax and indifferent, looking but little beyond the certificates indicating that the candidate has studied the term required by law; and the fact that he has the means of paying the necessary fees."

Whether this be true, or not, the county societies must be the best judges; we can only say, it is contrary to our experience. If the censors are qualified and faithful in the discharge of their duty, it is difficult to conceive that there could be such a discrepancy as to give rise to much injury. We certainly have heard of no complaint on this account. The favorite objection of the chairman is the *venality* of the examiners. The report says:

"Second, the *pay* of these various boards is made to depend entirely on the fees derived from the candidates, whom they license. They may find them but half qualified, and readily acknowledge they ought to be rejected. But, thinks the censor to himself, there are their certificates, showing that they have studied the time required by law, and if we refuse to license them, *we lose our day's work* besides our expenses, and after all, they are about as well qualified as some that are already in the profession. Hence we will give them a license, with some admonitions about further studies (as an anodyne for the conscience), and if the community do not like them they can refuse to employ them. That such thoughts and feelings do arise in the minds of the censors under the circumstances as mentioned, will be apparent to every one *who carefully notes his own mental operations.*"

We have no objection to the chairman expressing his own views, and giving his reasons, but we do protest against his setting up his own standard of moral rectitude as that of the profession. So far from such views being "apparent to every one," we believe that nine out of ten of those entrusted with the responsible duty of censor, would spurn with indignation the imputation that the paltry consideration of two or three dollars would make them unfaithful to their trust. Certainly in some, and we believe in many of the counties, the censors receive no compensation for their services; what then becomes of this slanderous imputation? It seems, the Medical Colleges are equally amenable to this charge; that they are even beset with stronger temptations, and are, if possible, more corrupt. The report says:

"If pecuniary considerations are brought thus to bear on the censors, they are no less direct and powerful on the medical colleges. The multiplicity of these institutions makes it necessary for them to make the most of every resource to sustain themselves, and the fee of twenty or twenty-five dollars from every graduate makes no small item in their income, particularly if they contrive to swell the list to twenty-five or thirty-five to each college annually. This, together with the natural desire on the part of the college to make as fair a show of prosperity as possible, and at the same time hold out as strong inducements as may be for the students to attend, contributes not a little to that laxity in examinations, which permits members annually to enter the profession, who have scarcely as much medical knowledge in their heads as is contained on the face of the parchment unworthily received from their Alma Mater."

As the above is unsustained by any evidence, it can only be received as the views of the chairman, and as indicative of what would be his own course under similar circumstances. We shall have occasion to show that the imputation is as groundless as that against the county censors, who receive no compensation for their services.

"Third—The present connection between the business of teaching and the power to license, is wrong in principle and injurious in practice. Such connection is therefore not only wrong in principle, but wholly *unphilosophical*. That it has been injurious in practice, the present state of the profession affords abundant evidence; indeed, such has been, and still is, the facility with which young men are converted into doctors through our professional tribunals, that it has grown into a proverb by no means destitute of truth, that there are more quacks *with* parchments than *without* them."

We believe that the practice has been almost universal throughout Christendom for literary institutions to confer their honors on their own students. It certainly prevails throughout Europe and in every state in this country. It was left for the chairman to make the discovery that it is "wrong in principle, and wholly unphilosophical." But this separation of the teaching from the licensing power is to be the sovereign panacea for all the ills the profession is heir to. We can recollect but one instance where the experiment was fairly tried, and we are surprised the chairman has not referred to it, if he did not recommend the precise act. Previous to the passage of the act of 1806, which is so obnoxious, and which it is now proposed to repeal, the powers of teaching and licensing were separate. Under this regulation the student was to be examined *before the governor, chancellor, judges of the supreme court, the attorney general, the mayor and the recorder of the city of New-*

*York, or any two of them, and their certificate constituted a license to practise.*

"Fourth—We object to the present arrangement, because it directs the attention of the licensing authority more to the time and place of study than to actual attainments."

As the resolutions do not touch this objection, or purpose any remedy, we shall give it no further notice.

"Finally—We object to the present system of medical education, because it tends to make superficial scholars, *and those only.*"

We have searched in vain throughout the resolutions for anything having the remotest tendency to correct this evil; on the contrary, we are satisfied the proposed changes would have a directly contrary effect. We cannot, however, resist the temptation to give the chairman's very graphic description of the present system of education; it is evidently a favorite theme, and one in which he revels with great delight. He says:

"The young man enters the office of a physician, pores over his library from day to day, gets now and then a question from his preceptor, occasionally sees a tooth extracted, or venesection performed, attends one or two courses of medical lectures, where the whole system of the theory and practice of medicine, materia medica, medical jurisprudence, obstetrics, surgery, physiology, pathology, and anatomy, are gone through with, in the space of sixteen weeks, a time scarcely sufficient to dissect profitably one subject, and to which is often attached hospital and clinical instruction; he is then honored with an M. D., and sent forth to kill or cure, as fortune may dictate."*

Again:—

"It is worse than folly to talk of quacks and quackery, to endow medical colleges, and organize medical societies, whilst whole scores are annually presented with the time-honored insignia of our profession, who are not only destitute of medical attainments, but also of the common rudiments of an English education, *and in moral character and mental capacity below the common dealers in lobelia, cayenne pepper and steam.*"

Such gross and wholesale charges may serve to round a period, may destroy or weaken the confidence of the community in the profession, and may seriously injure the reputation of our medical schools; but they can never form the basis of judicious reform. An honest lawyer never gives an opinion until he has examined his witnesses and investigated the merits of the case. A wise physician does not prescribe for his patient on vague rumors, but first ascertains whether his patient is sick, and the nature and character

* *Query.*—What Institution confers the degree of M. D. after a single course of lectures?

of the disease. How is it with our modern reformers in medicine? The chairman of the committee, N. S. Davis, has for four years held the office of CURATOR in the Medical Institution of Geneva College. It was his privilege, certainly, if not his duty, to have attended every examination, and voted on every candidate for a degree; he having as much voice in the recommendation as any professor in the institution. He had the legal right, and every facility was furnished for thoroughly examining the course of instruction; had he done so, we doubt whether he would have hazarded the assertions made in the report. *But he has never attended a single examination!* At the session of the State Medical Society, in 1835, one of the members, surprised at the statements made in the report, offered a resolution that the Censors of the State Medical Society attend the examinations at the respective Medical Colleges, and report to the society at its next meeting. This simple resolution to investigate the truth of the charges, *was opposed by the gentleman who made them, and rejected by the society.* Will the county societies sustain this crusade against their own institutions, when the State Society, which should have been the first to defend, refuses even to ascertain the truth or falsehood of the charges against them? We shall see.

Having very briefly examined some of the reasons offered in the report, we will proceed as briefly to examine the probable effect of the proposed changes. The effect which it would probably have on the county societies has been so ably depicted by the venerable Dr. Stearns in his appeal to the State Society, that we shall not add a word on that subject except to urge the perusal of his remarks, upon every one who takes an interest in the profession.

The period of study proposed in the first resolution is four years, the same as is required by the present law. If any student prefer the course recommended in the resolution he is at liberty to adopt it, and at the end of four years, if he can pass an examination, he can get his license. As early as 1818, some friends to improvement in the profession, believing that a better preliminary education and more general attendance on lectures would tend to elevate the profession, obtained the passage of a law, that one year's study of the branches usually taught in colleges, after the age of sixteen, or an attendance on one full course of lectures, should be received as a substitute for one year's study. The law had the effect desired by those who proposed it. Previous to its passage, very few students from the country ever attended lectures; now, it is very rare that a student offers himself for examination without having attended at least one course of lectures; most of them attend two, and many three. It is this feature of the law (which by many was thought to have operated so favorably) which it is proposed to repeal, and return back to the law as it was before 1818. Whether it would be an improvement, we leave for others to judge. Very few of the medical students in the country feel as if they could afford any greater expenditure of time or money than the law absolutely requires, and as nothing would be gained in time by an attendance on lectures, whilst the expenses would be materially increased, few, comparatively, would attend lectures. To suppose that this would, or could be corrected by the examinations, is an absurdity of which no one acquainted with medical examinations would be guilty. The mere amount of knowledge acquired by attendance on a single course of lectures is far from being the only advantage. The student obtains a plan for his whole course of study, learns what books to read, becomes acquainted with other students, forms more elevated conceptions of the dignity and usefulness of his profession, and acquires a spirit of emulation which lasts for life. Students who have attended one course of lectures (feeling the advantages they have derived) are always anxious to attend a second, and usually manage to accomplish their object* and those who have attended two courses are seldom content without obtaining a degree. Consequently many attend three and some four courses of lectures. The effect has been that comparatively few now obtain a County or State license. In 1835, the number of students reported as licensed by the State Censors, were *five;* by seventeen County societies that reported, *eight.* In 1846, the number reported by eighteen societies, was *eleven;* that of the State Censors was *seven*: amounting in all, for the year 1845, to *thirteen*, for 1846, to *eighteen*; whilst the number of graduates in the several Medical Schools in the State, averaged about *two hundred and twenty-five!* It is self evident that the object of the resolution was directed against the Medical Schools. Remove, as is proposed, the inducement to attend the first course of lectures and take away the power of obtaining a degree with the stimulus which it imparts, and if the effect is not retrograde there is neither force in reason, nor truth in past experience. The directions in the resolutions as to what the Censors shall require, are almost too puerile to demand attention. We can only say, that a Censor that requires such directions would be totally unfit for his office. Who is to determine what constitutes "*a good academical education?*" There is, however, one requirement we cannot pass over in silence. The student must give evidence of having "assisted in the dissection of two subjects." With due deference to the State Society, we do not think it quite equal to the proposed requirements of the Thomsonians, which required that every candidate should give evidence of having treated at least two cases successfully. Many a tyro knows to his sorrow, that, without proper instruction, he may assist in the dissection of a dozen subjects without becoming an anatomist. A committee of the Massachusetts Medical Society, composed of John C. Warren, John Dixwell, and George Hayward,

* Dr. Davis, we think, stated at the National Medical Convention that he had attended three courses, although he had to board himself at the expense of twenty-five cents a week. This shows Dr. D.'s estimate of the value of Medical Schools. Since that time, however, they have much improved.—[*Ed. N. Y. Jonrnal.*]

—men who are the ornaments and pride of our profession, seem to estimate differently the attendance on medical lectures. They say:—

"The counsellors of this Society consider courses of lectures to be indispensible to an adequate knowledge of the medical art, and, being aware that students acquire much more information from a second course, than from a first, do therefore concur in this third proposal, and in the amendment made by the New Hampshire Medical Society, which substitutes two courses of public Medical Lectures in some legally organized and respectable school, for one course."

What possible advantage does the proposed organization of a Board of Censors have over the present Board of State Censors? We can see none. Whilst the present Boards of County Censors possess many advantages over the proposed mode, they are simple, cheap, convenient for the student,—would be far better acquainted with the character and habits of the candidates, and equally efficient with the proposed Board. The contemplated organization on the contrary, is complicated, inconvenient for both pupils and censors, and expensive.

We come next to the consideration of the probable effect on the medical schools. We have already seen that the tendency would be to prevent our own students from attending lectures; but the deservedly high reputation of our schools attracts many pupils from other States, and from Canada. It has been estimated that one-half our students were of this class; perhaps one-third would be a nearer estimate. Take from our coleges, as is proposed, the power of conferring degrees, and you at once cut off the whole class of foreign students; deduct also the probably diminished numbers of our own students, and our medical schools may close their doors. We are credibly informed that Dr. Davis declares that he has no hostility to our medical schools; of course we are bound to believe him; but they may well exclaim: "Save us from such friends — our worst enemy could not have done us greater wrong." Having attempted to show the reasons offered in the report for the proposed change, the probable effect upon the profession and our medical institutions, we shall close by very briefly describing the mode of examination and requirements in one of our medical institutions, to see how far it is amenable to the charges or statements made in the report. In the first place, the State law requires that the candidate should be 21 years of age, should have studied medicine three years with some regularly licensed physician, and have attended two full courses of lectures in regularly incorporated medical institutions. He is required to furnish a dissertation on some medical subject, composed and written by himself, which is examined and approved by the professors, before the candidate is admitted to an examination, Each student is examined seperately by the professors, in the presence of the whole board of professors; if approved, he is reccommended to present himself before the Joint Board of Faculty and Curators, upon whom rests the responsibility of recommending him to the Trustees for a degree. This Board is composed of six professors and forty curators; six of the latter (equal to the whole faculty) are appointed by the State Society, the remainder are appointed by the Trustees, and selected from the most respectable and intelligent of the profession. The students are thoroughly examined in the presence of this Board, each *member having an equal vote in the recommendation:* if requested, dissertations are read and defended in the presence of the Curators. The number of Curators that attend and vote, usually varies from ten to fifteen; the recommendation of at least three Curators is absolutely necessary to enable the Trustees to confer a degree. *Not one cent of the fee for diplomas goes into the pockets of the professors, neither do they receive any compensation for the labor and trouble of examination whether the number be five or fifty.* The course of instruction has ever been open to investigation; the Faculty have not only courted, but have solicited investigation, and they fearlessly appeal to their graduates, and the Curators who have attended the examinations against the slanders of the report, so far as they were intended to apply to Geneva College.

All will admit that there are defects in our system of medical education, but we deny that they are half as great or glaring as represented in the report. We believe that the medical profession of the State of New-York will not suffer in comparison with that of any other State in the Union, or of any other country, in the practical acquirements of the profession; and we have reason to know that our Medical Schools are as strict in their requirements, and in the qualifications of their graduates, as those of the neighboring States. Even the chairman is compelled to admit that the greatest defects consist in the want of sufficient *preliminary education and defective office instruction;* but for *these defects* we search in vain for any proposed remedy, either in the resolutions or report. These are defects, not in the system, but in its practical operation, and would be quite as likely to exist under the proposed system, with all its clumsy machinery, as under the present system.

